# Knowledge, attitudes, and practice of female genital mutilation and cutting: an observational cross-sectional study in English primary care (FGM/C Study)

**DOI:** 10.3399/BJGPO.2023.0005

**Published:** 2023-07-12

**Authors:** Michelle Elizabeth Robinson, James Prior, Christian D Mallen, Thomas A Shepherd

**Affiliations:** 1 School of Medicine, Keele University, Keele, UK; 2 Research and Innovation Department, St George’s Hospital, Midlands Partnership NHS Foundation Trust, Stafford, UK

**Keywords:** primary health care, FGM, circumcision, female, general practice

## Abstract

**Background:**

Female genital mutilation and cutting (FGM/C) describes procedures involving partial or total removal of the external female genitalia or other injury to the female genital organs for non-medical reasons. Increasing migration means many communities living in the UK originate from countries where FGM/C is practised. Consequently, clinicians in the UK are increasingly exposed to women and children who have experienced FGM/C.

**Aim:**

To explore the knowledge, attitudes, and practice of primary care GPs and practice nurses (PNs) regarding FGM/C.

**Design & setting:**

An observational cross-sectional study with GPs and PNs working in primary care in the West Midlands, UK.

**Method:**

An online survey was circulated to GPs and PNs between September 2019 and December 2019.

**Results:**

A total of 137 survey responses were received. Study participants were predominantly female (81.8%) and GPs (59.9%), with a mean age of 47.3 years (standard deviation [SD] 9.1). The survey found 19.7% of responders reported seeing >1 patient with FGM/C in the past 12 months. It also found 91.3% of responders had received some form of FGM/C training; however, the format and frequency of training varied and 34.3% felt they had received inadequate training to manage treatment of FGM/C.

**Conclusion:**

The results have suggested varying degrees of competence and confidence associated with recognising and managing patients with FGM/C in primary care in the West Midlands. Given that patients with FGM/C typically present in primary care, it is important that clinicians can provide appropriate support underpinned by up-to-date training.

## How this fits in

The study explored the knowledge, attitudes, and practice of primary care practitioners in the management of FGM/C. Global migration means that there are increasing levels of patients with FGM/C presenting in primary care. Training for GPs is mandatory in the UK; however, there is clearly a wide variation in the frequency of contact with patients in primary care having undergone FGM/C. Results suggest varying degrees of confidence and competency in practitioners, which has implications for training provision and emphasises the importance of training delivered to other healthcare professionals in primary care.

## Introduction

FGM/C describes procedures involving partial or total removal of the external female genitalia or other injury to the female genital organs for non-medical reasons.^1^ There are four main types of FGM/C, which are defined by the World Health Organization (WHO) as the following: type 1, which is the partial or total removal of the clitoral glans (the external and visible part of the clitoris, which is a sensitive part of the female genitals), and/or the prepuce or clitoral hood (the fold of skin surrounding the clitoral glans); type 2, which is the partial or total removal of the clitoral glans and the labia minora (the inner folds of the vulva), with or without removal of the labia majora (the outer folds of skin of the vulva); type 3, is also known as infibulation, which is the narrowing of the vaginal opening through the creation of a covering seal. The seal is formed by cutting and repositioning the labia minora or labia majora, sometimes through stitching, with or without removal of the clitoral prepuce or clitoral hood and glans; and type 4, which is all other harmful procedures to the female genitalia for non-medical purposes; for example, pricking, piercing, incising, scraping, and cauterising the genital area.^2^

Over 200 million girls and women alive today have been subjected to the practice, with more than 3 million girls estimated to be at risk of FGM/C annually.^2^ Large-scale representative surveys show that the practice of FGM/C is highly concentrated in a swath of countries from the Atlantic coast to the Horn of Africa, in areas of the Middle East, such as Iraq and Yemen, and in some countries in Asia (including Indonesia), with wide variations in prevalence.^3^

Increasing global migration means there are many communities living in the UK who originate from countries where FGM/C is practised and so clinicians in the UK are increasingly exposed to women and girls who have experienced FGM/C.^4^ An estimated 137 000 women and girls with FGM/C, born in countries where FGM/C is practised, were permanently residing in England and Wales in 2011. This represented a prevalence rate of 4.8 per 1000 population.^5^ A health economics report commissioned by the Department of Health estimated that the annual cost of care for women and girls with FGM/C in England and Wales is £100 million; a significant proportion of which is either unmet needs or non-recurrent. Provision of services to support women and girls with FGM/C will reduce the need for services, thus reduce this figure. Investment in good FGM/C services leads to long-term significant savings to the NHS.^6^

FGM/C is typically performed during childhood or adolescence and is therefore relevant to paediatric services. However, owing to the wide range of physical and psychological consequences of FGM/C, such as shock, post-traumatic stress disorder (PTSD), infection, and problems related to sexual health, patients often present in primary care.^7,8^ Training for GPs on FGM/C is mandatory in the UK, and information is readily available from the Royal College of General Practitioners^9^ and the Royal College of Nursing.^10^ There is, however, wide variation in the frequency of contact with patients in primary care having undergone FGM/C. This study aimed to explore the knowledge, attitudes, and practice of healthcare professionals in primary care who are treating and managing patients with FGM/C.

## Method

### Study population

One hundred and thirty-seven GPs and PNs from 19 clinical commissioning groups (CCGs) in the West Midlands were recruited via email through the National Institute for Health and Care Research Clinical Research Network (CRN). An invitation email was sent to GPs and PNs that contained a link to a participant information sheet, online consent form, and online survey. To be eligible to take part in the study, GPs and PNs needed to be working within primary care in the West Midlands.

### Study design and procedures

An observational cross-sectional online survey was circulated to GPs and PNs from 20 CCGs in the West Midlands (no submissions were received from one CCG). To increase response to the survey, reminder emails were requested to be sent after 2 weeks and 4 weeks, and additionally some areas chose to raise awareness of the survey among practice managers to facilitate its completion.

The content of the survey was based on topics and findings from previously published work examining knowledge, attitudes, and practice regarding FGM/C.^6,11,12^ Domains included questions about FGM/C training and experience, and confidence in their knowledge of FGM/C. Additionally, baseline demographics, such as age, sex, role, and years in practice, were collected. The survey was designed using an online survey builder, Jotform. Survey content underwent a process of review and refinement during the development of the survey with members of the research team to ensure its ease of use and that it was possible to complete within the timeframe. Suggestions from the testing process were then incorporated into the final survey.

Analysis was performed using Microsoft Excel. Frequency tables were used to collate responders' survey data.

## Results

A total of 137 surveys were completed between September 2019 and December 2019. Table 1 illustrates the baseline characteristics of responders. Study participants were predominantly female (81.8%) and GPs (59.9%). Responders had a mean age of 47.3 years (SD 9.1) and a mean of 23.0 years’ experience (SD 10.5).

**Table 1.** Baseline characteristics (*N* = 137)

Variable	*n* (%)^a^
**Age, years**	
18–30	3 (2.2)
31–40	30 (21.9)
41–50	49 (35.8)
<50	59 (43.1)
Mean (SD)	47.3 (9.1)
**Sex**	
Male	25 (18.2)
Female	112 (81.8)
**Years in practice**	
<5	5 (3.6)
5–10	13 (9.5)
11–20	44 (32.1)
21–30	40 (29.2)
>30	35 (25.5)
Mean (SD)	23.0 (10.5)
**Healthcare profession**	
GP	82 (59.9)
Practice nurse	33 (24.1)
GP registrar or physician associate^b^	4 (2.9)
Advanced nurse practitioner or nurse practitioner^b^	13 (9.5)
Other	5 (3.6)

^a^Unless otherwise stated. ^b^Owing to small numbers in some groups these have been collapsed. FGM/C = female genital mutilation/cutting. SD = standard deviation.

Table 2 illustrates responders experience with FGM/C. The survey found 73.0% of responders reported not seeing any patients who had undergone FGM/C in the past 12 months; 51.1% of responders had received FGM/C training within the past 12 months, while 8.8% reported never having received any training on FGM/C. The FGM/C training received was predominantly face to face with no formal assessment (51.2%). It also found 48.2% of responders felt that they had received adequate training to manage treatment of uncomplicated FGM/C cases, whereas 34.3% felt they had inadequate training to manage treatment of FGM/C. In addition, 38.7% of responders said that the GP was the main continuing point of contact for patients with FGM/C, while 27.0% did not know who the continuing point of contact was for their practice.

**Table 2.** Training and experience with FGM/C (*N* = 137)

Category	*n* (%)
**Number of patients treated who have undergone FGM/C in the past 12 months**	
0	100 (73.0)
1–5	17 (12.4)
6–10	6 (4.4)
11–15	1 (0.7)
16–20	1 (0.7)
>20	2 (1.5)
Missing	10 (7.3)
**When FGM/C training was last received**	
Within the past 6 months	22 (16.1)
6–12 months ago	48 (35.0)
1–2 years ago	40 (29.2)
3–5 years ago	12 (8.8)
>5 years ago	3 (2.2)
Never received training on FGM/C	12 (8.8)
**Type of FGM/C training received^a^**	
Day or weekend face-to-face courses with no formal assessment	64 (51.2)
Online course or module with no formal assessment	58 (46.4)
Courses or modules with formal assessments (exams, marked assignments, and so on)	7 (5.6)
Other	10 (8.0)
**Adequate training received to manage patients who have undergone FGM/C**	
I feel I have had **adequate** training to manage treatment of **complex** FGM/C cases	7 (5.1)
I feel I have had **adequate** training to manage treatment of **uncomplicated** FGM/C cases	66 (48.2)
I feel I have **inadequate** training to manage treatment of FGM/C	47 (34.3)
I feel I have **no training** to manage treatment of FGM/C	17 (12.4)
**Who is the main, continuing point of contact for patients with FGM/C**	
GP	53 (38.7)
Practice nurse	9 (6.6)
Patients are referred to specialist care	22 (16.1)
Other	15 (10.9)
Don’t know	37 (27.0)
Missing	1 (0.7)

^a^ *N* = 125. FGM/C = female genital mutilation/cutting.

Responders were asked to rate on a scale of 0–10 (0 being not at all confident and 10 being completely confident) several statements relating to their confidence in their FGM/C knowledge. The results are presented in Table 3. Responders rated their confidence higher for knowing what to do if they believed a child was at risk of FGM/C (mean 6.8), also for identifying patients from communities most at risk of FGM/C (mean 6.3). Responders rated their confidence lower in identifying that FGM/C type 3 and type 4 had occurred during an examination (mean 4.8 and 4.2, respectively). Responders rated their confidence lower in knowing what support could be offered to patients who had experienced FGM/C (mean 4.6).

**Table 3.** Healthcare professional confidence in FGM/C knowledge

Variable	Mean^a^	SD	Missing, %
Identifying patients from communities most at risk of FGM/C	6.3	2.3	0.7
Discussing FGM/C with a patient consulting with symptoms suggesting FGM/C	5.8	2.6	1.5
Identifying that FGM/C type 1 (clitoridectomy) has occurred during an examination	5.1	3.1	0.7
Identifying that FGM/C type 2 (excision) has occurred during an examination	5.1	3.2	1.5
Identifying that FGM/C type 3 (infibulation) has occurred during an examination	4.8	3.4	0.0
Identifying that FGM/C type 4 (pricking, piercing, incising, scraping, and cauterising) has occurred during an examination	4.2	2.9	0.0
Recognising the short-term complications of FGM/C	5.5	2.7	0.0
Recognising the long-term complications of FGM/C	5.5	2.7	1.5
Knowing what psychiatric syndromes a patient could have as a result of FGM/C	5.0	2.9	0.7
Knowing what to do if you believe that a child may be at risk of FGM/C	6.8	2.7	0.0
Knowing what support can be offered to patients who have experienced FGM/C	4.6	2.8	0.7

^a^Scale 0–10; 0 = not at all confident, 10 = completely confident. FGM/C = female genital mutilation/cutting. SD = standard deviation.

## Discussion

### Summary

This study aimed to explore the knowledge, attitudes, and practice of primary care clinicians regarding FGM/C in the West Midlands. Key findings from the survey showed that 19.7% of survey responders had seen patients in primary care in the past 12 months who had undergone FGM/C.

A high percentage of responders had received some form of FGM/C training (91.3%); however, the format and frequency of when they had last received training varied and 34.3% felt they had received inadequate training to manage treatment of FGM/C, suggesting varying degrees of competency associated with recognising and managing FGM/C.

Results of this research have suggested that improvements could be made in how patients who have undergone FGM/C are managed in primary care.

### Strengths and limitations

The authors acknowledge that this study only represented a small number of healthcare professionals in the West Midlands and are unable to make any generalisations about rates of FGM/C in the UK. Responses were received from 19 out of 20 CCGs in the West Midlands, so the sample should be representative of the West Midlands, but not necessarily of the UK more generally. It is also possible that patients who had experienced FGM/C could have consulted but were not identified by the healthcare professional, additionally there could be inaccuracies with survey responders' recall of how many FGM/C patients they had seen over the 12-month period.

It was aimed to distribute the survey to healthcare professionals in the most efficient way and to reduce their burden in completing the survey, as GPs are typically difficult to recruit for research.^13^ The survey was distributed through CRNs to capture a larger number of responses and is a method that has been used previously. However, this approach meant that it wasn’t possible to calculate a true response rate to the survey and data were unable to be collected on non-responders. Another limitation is that the survey was short to minimise survey completion time and the burden of participation, to aid with recruitment. Other data collection measures that went beyond practitioner confidence ratings, including care and referral pathways used by practitioners, could have added depth to the findings. A high proportion of females completed the survey compared with males and so mechanisms to increase male representation in this type of research should be explored as well as ways to enhance recruitment more generally, such as using social media or other GP and PN networks or offering incentives for survey completion.^14^

### Comparison with existing literature

Healthcare professionals who responded to the survey had lower confidence around knowing what support can be offered to patients who had experienced FGM/C. It is known from previous research that patients with FGM/C can require support from mental and sexual health services.^15,16^ GPs' and PNs’ main role can be of identification,^9^ but this needs to be underpinned by correct, up-to-date training and support to be able to help such patients. First, by improvements in being able to recognise the four types of FGM/C to be able to diagnose that a patient has experienced FGM/C. The present research is consistent with a 2013 study in London that showed healthcare professionals surveyed had insufficient knowledge about the diagnosis and classification of FMC/C.^17^ A 2020 qualitative study exploring perspectives of GPs in England found that recent focus on FGM/C and safeguarding was helpful in raising awareness, it was only a relatively recent part of formal education, and that seeing a patient with FGM/C could be both complex and stressful. The researchers heard concerns about the risks of offending women, breaching cultural sensitivities, or raising memories of a potentially traumatic experience. FGM/C is experienced as complex to manage in primary care, with challenges including consideration of how and when to talk about FGM/C, and how to meet the potential needs of both the woman who presents and her family. Managing FGM/C reporting and recording brings additional tensions into the consultation.^18,19^

Healthcare professional training requirements around this area are complex and the demand for adequate FGM/C training for UK healthcare professionals will only grow. Although FGM has declined globally over the past 30 years, prevalence remains high owing to population growth. Therefore, if trends continue, the number of girls and women undergoing FGM will rise significantly in the next 15 years, in turn raising national healthcare cost of care.^20^ In addition, the effects that the recent COVID-19 pandemic may have on FGM/C globally are still being realised. Estimates provided by Avenir Health, Johns Hopkins University, and Victoria University predicted that significant levels of lockdown-related disruption over 6 months may have caused considerable delays in programmes to end FGM/C, potentially leading to around 2 million more cases of FGM/C over the next decade than would otherwise have occurred.^21^ Therefore, additional resources are urgently needed to scale-up interventions that can prevent FGM in the future and reduce health complications.

The WHO have launched a training manual on person-centred communication (PCC), a counselling approach that encourages healthcare providers to challenge their FGM-related attitudes and build their communication skills to effectively provide FGM prevention counselling,^2^ further research that investigates the efficacy of this training, and if this training approach can improve healthcare professional knowledge, attitudes, and practice of FGM/C could be explored further.

### Implications for research and practice

In conclusion, given that patients with FGM/C typically present in primary care in the UK, it is critical that clinicians are given the adequate training required to provide appropriate care for patients presenting with FGM/C. In addition, it should be considered that training be delivered to other relevant healthcare professionals, given that treatment and management are often by those in other primary care roles such as PNs. Training needs to not only satisfy clinical requirements, but also enable healthcare professionals to feel competent in the recognition and management of patients who have experienced FGM/C, and to contribute to FGM prevention. Future research needs to focus on evaluating training to improve healthcare professionals' confidence, particularly around identifying type 3 and type 4 FGM/C, as well as the psychiatric syndromes patients who have experienced FGM/C may experience.

## Data

The dataset relied on in this article is available from the corresponding author on reasonable request.
